# Mouse mammary tumor virus-like gene sequences are present in lung patient specimens

**DOI:** 10.1186/1743-422X-8-451

**Published:** 2011-09-24

**Authors:** Laura M Trejo-Avila, Pablo Zapata-Benavides, Raúl Barrera-Rodríguez, Isaías Badillo-Almaráz, Santiago Saavedra-Alonso, Diana E Zamora-Avila, Karla Morán-Santibañez, Jorge A Garza-Sáenz, Reyes Tamez-Guerra, Cristina Rodríguez-Padilla

**Affiliations:** 1 Departamento de Microbiología e Inmunología, Facultad de Ciencias Biológicas, Universidad Autónoma de Nuevo León (UANL). Ave. Universidad S/N. Ciudad Universitaria, San Nicolás de los Garza, Nuevo León, 66451, Mexico; 2 Departamento de Bioquímica. Instituto Nacional de Enfermedades Respiratorias-SSA México. Calzada de Tlalpan No.4502. Col. Sección XVI, D.F. 14080, Mexico; 3 Hospital Regional de Zacatecas. Guerrero 116, Col. Centro, Zacatecas, Zacatecas, 98000, Mexico

**Keywords:** MMTV, lung cancer, Mexico

## Abstract

**Background:**

Previous studies have reported on the presence of Murine Mammary Tumor Virus (MMTV)-like gene sequences in human cancer tissue specimens. Here, we search for MMTV-like gene sequences in lung diseases including carcinomas specimens from a Mexican population. This study was based on our previous study reporting that the INER51 lung cancer cell line, from a pleural effusion of a Mexican patient, contains MMTV-like *env *gene sequences.

**Results:**

The MMTV-like *env *gene sequences have been detected in three out of 18 specimens studied, by PCR using a specific set of MMTV-like primers. The three identified MMTV-like gene sequences, which were assigned as INER6, HZ101, and HZ14, were 99%, 98%, and 97% homologous, respectively, as compared to GenBank sequence accession number AY161347. The INER6 and HZ-101 samples were isolated from lung cancer specimens, and the HZ-14 was isolated from an acute inflammatory lung infiltrate sample. Two of the *env *sequences exhibited disruption of the reading frame due to mutations.

**Conclusion:**

In summary, we identified the presence of MMTV-like gene sequences in 2 out of 11 (18%) of the lung carcinomas and 1 out of 7 (14%) of acute inflamatory lung infiltrate specimens studied of a Mexican Population.

## Background

Lung cancer is the most common type of cancer worldwide; it has the highest prevalence and mortality rates in Mexico, and the death rate is increasing [1,2]. There are several risk factors for developing lung cancer; however, smoking is the major risk factor. In countries with a high prevalence of smoking, approximately 90% of the lung cancer diagnoses are attributable to cigarette smoking [3]. Other cases are attributable to occupational exposure to lung carcinogens, such as arsenic, asbestos, beryllium, cadmium, chromium, diesel fumes, nickel, and silica[4-7]. Recently, a viral etiology was proposed, because sequences for gene viral products were detected in patients with lung cancer[8]. Previous studies have reported that human Papillomavirus (HPV) infection may be related to pulmonary adenocarcinoma tumorigenesis[9]. The predominant genotype identified was HPV 16, followed by HPV 18, [10,11] and it has been reported that HPV 16/18 infection is associated with non-smoking Taiwanese female lung cancer[11]. Other studies reported the detection of have detected Epstein-Barr virus in adenocarcinomas and Squamous cell lung cancer[12,13]. Moreover, zoonotic viruses, such as Jaagsiekte sheep retrovirus, have been identified in sheep breeders who develop lung cancer[14].

Recently, Murine Mammary Tumor Virus (MMTV)-like env gene sequences have been identified in humans and are associated with breast carcinoma [15-18]. The whole proviral structure, which shares 95% homology with MMTV, was identified in two human breast cancers and was designated as Human Mammary Tumor Virus (HMTV) [18]. An epidemiological study on a United States population identified MMTV-like gene sequences in 38% of breast cancer tissue specimens, as compared to <2% in normal breast tissue specimens[16]. The prevalence of MMTV-like gene sequences is: 38% in North America;[16,17] 38% in Italy;[19,20] 38% in Australia;[21] 31% in Argentina;[17] 74% in Tunisia;[20] 16.8% in China;[22] and 4.2% in Mexico[23]. Our group previously identified MMTV-like gene sequences in the INER51 lung cancer cell line, suggesting that these sequences may exist in other tumor types[23]. Here, we extend the search for MMTV-like gene sequences in a Mexican population diagnosed with lung cancer and acute inflammatory lung infiltrate.

## Results

### Amplification of MMTV *env *sequences from INER51

**Figure 1:**
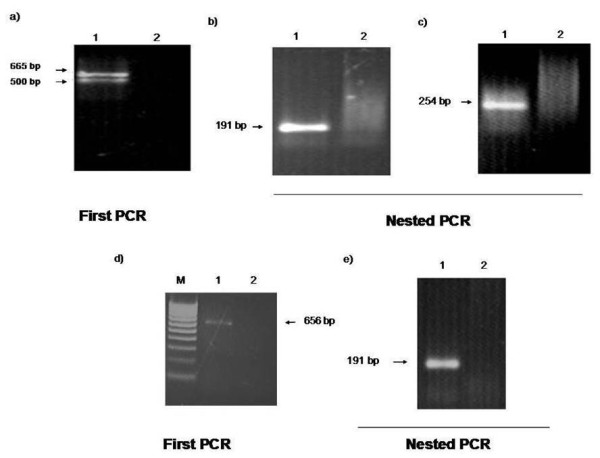
**PCR Amplification of MMTV-like *env *gene sequences in the INER51 cell line**. a) The first PCR reaction used the 1-3 primer sets to amplify a 665-bp product. The DNA was re-amplified using b) the 2N-3L primers to amplify a 191-bp product and c) the 2N-3N primers to amplify a 254-bp product. d) We used the 5L-3N primers to amplify a 656-bp product. The DNA was re-amplified e) using 2N-3L primers to yield a 191-bp product.

First, we analyzed the DNA from the INER51 lung cancer cell line using primers 1-3 and 5L - 3N. We re-amplified the DNA using primers 2N - 3L and 2N - 3N, as shown in Figure 1. All PCR assays yielded bands with the expected product size, except primers 1-3. This primer set amplified two products: one was 665-bp (expected product), and the other was 500-bp. INER51was used as a positive control for all subsequent assays.

### Amplification of *env*, LTR, and *gag *MMTV-like sequences from lung cancer samples

**Figure 2:**
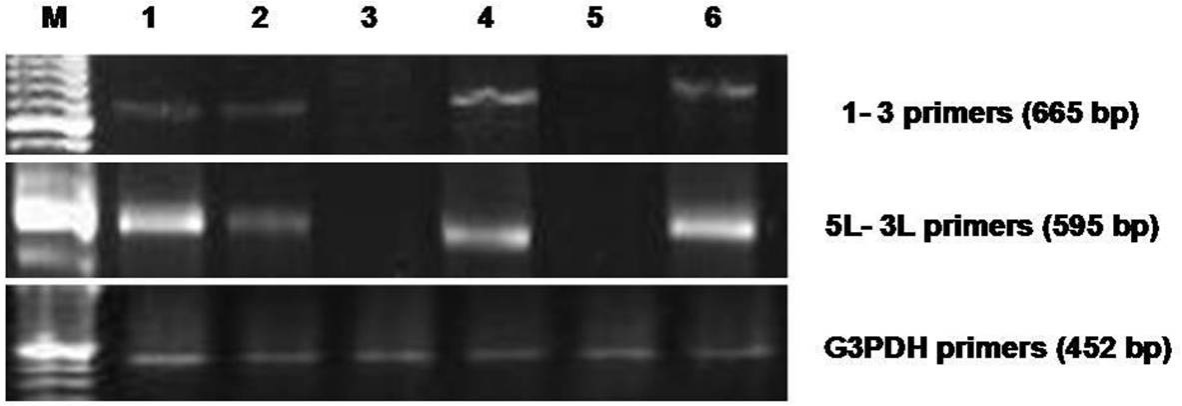
**Amplification of MMTV-like *env *gene sequences in lung samples**. Agarose gel electrophoresis of the 665-bp PCR product using primers 1-3 (top panel). The 595-bp PCR product using the 5L-3L primers (middle panel). As a PCR control, we used G3PDH primers to amplify a 452-bp product (bottom panel). Lane M contains a marker; lane 1 is INER51; lane 2 is INER6; lane 3 is IN-9; lane 4 is HZ-14; lane 5 is HZ-19; and lane 6 is HZ-101.

**Figure 3:**
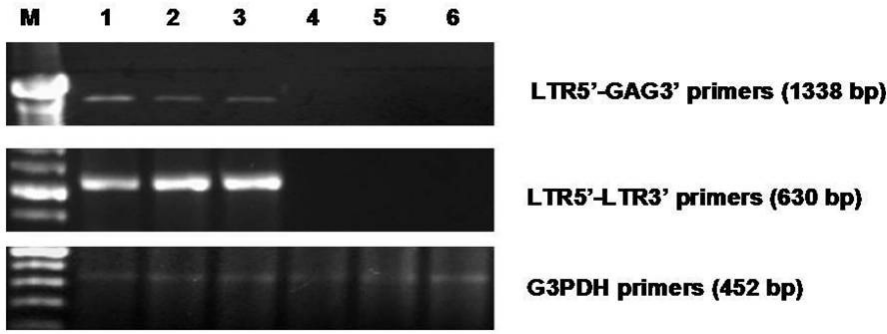
**Amplification of *ltr*-gag sequences in lung samples**. Agarose gel electrophoresis of the 1338-bp PCR product using LTR5'-GAG3' primers (top panel). The 620-bp PCR product was amplified using the LTR5'-LTR3' primers (middle panel). As a PCR control, we used G3PDH primers to amplify a 452-bp product (bottom panel). Lane M contains a marker; lane 1 is INER51; lane 2 is IN6; lane 3 is HZ14; lane 4 is HZ101; lane 5 is HZ-19; and lane 6 is the negative control.

We analyzed 11 lung cancer samples and seven specimens with other lung pathologies (Table 1). The lung samples were amplified with primers 1-3, and nested PCR was performed using primers 5L-3L. In both PCR tests, the sequences were amplified in three samples, as shown in figure 2. To confirm the presence of the MMTV-like gene sequences.in the lung samples, the three positive samples were amplified using primers of a LTR-*gag *region as shown in figure 3.

**Table 1:** Pathology of lung tissues samples included in the study

Sample	Type	Diagnosis
**HZ 27**	Biopsy	Mediastinal lymphoma
**HZ 28**	Biopsy	Infiltrating adenocarcinoma
**HZ 40**	Biopsy	Infiltrating adenocarcinoma
**HZ 101**	Bronchial washing	Bronchogenic carcinoma
**HZ 106**	Bronchial washing	Bronchogenic carcinoma
**IN 03**	Bronchial washing	Adenocarcinoma
**IN 06**	Bronchial washing	Adenocarcinoma
**IN 09**	Bronchial washing	Adenocarcinoma
**IN 11**	Bronchial washing	Micropapillary adenocarcinoma
**IN 12**	Bronchial washing	Micropapillary adenocarcinoma
**IN 14**	Bronchial washing	Lung cancer pulmonary anthracosis
**HZ 05**	Biopsy	Acute pulmonary inflammatory infiltrate
**HZ 10**	Biopsy	Acute pulmonary inflammatory infíltrate
**HZ 14**	Biopsy	Acute pulmonary inflammatory infíltrate
**HZ 16**	Biopsy	Acute pulmonary inflammatory infíltrate
**HZ 17**	Biopsy	Acute pulmonary inflammatory infíltrate
**HZ 32**	Biopsy	Acute pulmonary inflammatory infíltrate
**HZ 42**	Biopsy	Acute pulmonary inflammatory infiltrate

To discard any possible contamination in the amplifications, PCR reactions were run with reagents and laboratory facilities where they never have worked with cell culture or with MMTV (Laboratory of Genetics, Facultad de Medicina Veterinaria y Zootecnia UANL) performing here the whole process of samples processing.

Sequencing was performed to confirm the identities of the MMTV-like sequences of the PCR products using the 5L-3L primers. The reported nucleotide sequence data are available in the DDBJ/EMBL/GenBank databases under accession numbers: GU252129 (INER6), HM636471 (HZ-101), and HM636470 (HZ-14).

**Figure 4:**
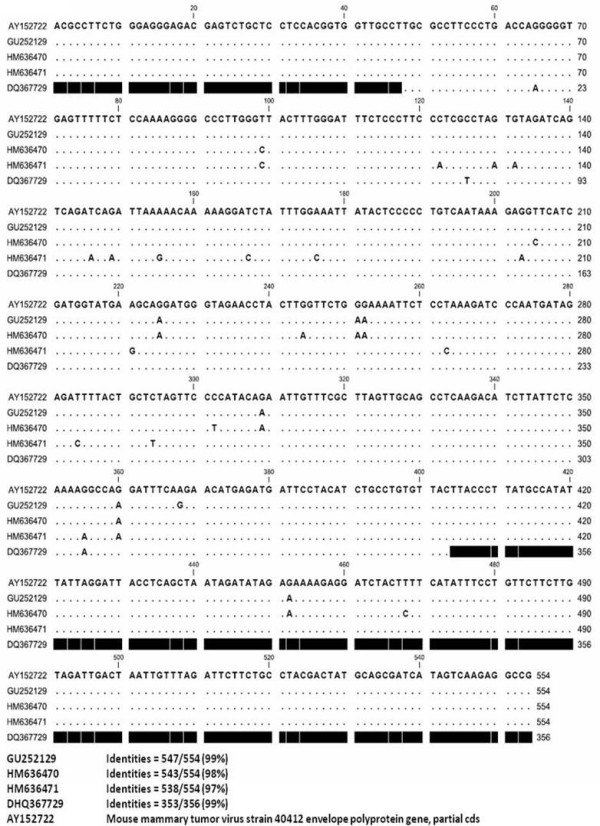
**MMTV-like *env *gene sequencing**. Comparison of *env *gene sequences amplified from INER6 (GU252129), HZ-101 (HM636471), HZ-14 (HM636470), and INER51 (DQ367729).

The INER6 and HZ-101 samples were isolated from lung cancer specimens, and the HZ-14 was isolated from an acute lung inflammation infiltrate specimen sample. The homology of the INER6, HZ-101, and HZ-14 sequences of the MMTV env region were 99%, 98%, and 97% respectively, as compared to Gen Bank accession number AY152722. The INER6 and HZ-101 sequences were mutated in their reading frame, resulting in nonsense mutations (Figure 4).

## Discussion

Lung cancer has a worldwide distribution, and multiple risk factors have been identified. The main risk factor is smoking; however, lung cancer has been identified in non-smoking populations. Additional factors that may influence the development of lung cancer are viruses; viral gene sequences corresponding to HPV,[8] EB,[12] Jaagsiekte sheep retrovirus have been identified in patients with lung cancer [14] and more recently MMTV have been identified in a lung cancer cell line[23].

A high prevalence of MMTV was reported for breast cancer patients in the United States (38%) and Argentina (31.8%); however, the prevalence was 4.2% in Mexico. The detection method used in USA and Argentina specimens was more sensitive than one used with Mexican samples. It could be the explanation of the different between them [15,24]. Previous studies report on the presence of viruses in patients who have other malignancies [25]. In a previous paper, we reported on the presence of MMTV-like gene sequences in the INER51 lung cancer cell line. This cell line was established from a pleural effusion of a patient diagnosed with primary lung cancer by The INER-SSA in Mexico City[23]. The cell line was used as a positive control.

In this paper, we report the presence of MMTV-like gene sequences in 2 lung carcinomas and a acute inflammatory lung infiltrate samples that were positive for MMTV when analyzed using the 1-3 and 5L-3L primers. We amplified the MMTV-like LTR-gag region using the primers reported by Liu et al to confirmed their positivity [18] Furthermore, we used the INER51 cell line as a positive control. In 2010, Johal H et al.[25] reported on the detection of MMTV-like *env *sequences in ovarian, prostate, endometrial, and skin cancers, but not in lung cancer, indicating that MMTV-like presence is not restricted to breast cancer cells. We detected MMTV-like gene *env *sequences in the INER51 lung cancer cell line [23]. Here, we show that MMTV-like gene sequences exist in lung samples from a Mexican population and support that the presence of MMTV-like sequences is not restricted to breast cancer cells.

A very important aspect to consider is that samples processing an PCR reactions were made also in Laboratory of Genetics of Facultad de Medicina Veterinaria y Zootecnia UANL, where people have never worked with cell lines (including INER51) or MMTV genetic material and therefore the risk of DNA contamination is null.

In this study, we analyzed the MMTV-*env *sequences in two lung cancer samples and the results suggested that nonsense mutations were caused by deamination (TGG to TGA or TGG to TAG). Human APOBEC3G (APOBEC-related cytidine deaminase, hA3G) deaminates cytosine residues within single-stranded DNA during reverse transcription, resulting in high levels of plus-strand G-to-A mutations [26]. Therefore, hA3G can introduce nonsense mutations, such as TAG or TGA, in the plus-strand coding sequence, since TGG is a target of hA3G. Consistent with this finding, it was reported that most nonsense mutations in the HTLV-1 proviruses in cases of adult T-cell leukemia were caused by deamination [27].

## Conclusions

In this study, we detected MMTV-like *env *gene sequences in three out of 18 lung tissues specimens obtained from Mexican patients. Two samples assigned as INER6 and HZ-101 were isolated from lung cancer specimens, and the HZ-14 sample was isolated from an acute inflammatory lung infiltrate sample. The three identified MMTV-like gene sequences were 99%, 98%, and 97% homologous, respectively, as compared to GenBank sequence accession number AY161347. Two of the *env *sequences exhibited disruption of the reading frame suggesting that nonsense mutations were caused by deamination (TGG to TGA or TGG to TAG).

## Methods

### Cell Line and Tissue Samples

Eleven lung cancer samples and seven samples of other lung pathologies, including pulmonary anthracosis and acute pulmonary inflammation infiltrate (Table 1), were obtained from the Hospital Regional de Zacatecas in Mexico City and the Instituto Nacional de Enfermedades Respiratorias (INER) in Mexico City. The non-small cell lung cancer cell line INER51 was established and obtained from the Instituto

Nacional de Enfermedades Respiratorias-SSA, Mexico City, from the pleural effusion of a patient diagnosed with primary lung [28]. This cell line was maintained in DMEM/F-12 with 10% fetal bovine serum (FBS) in (5%CO2) at 37°C.

### DNA Isolation

DNA was extracted from tumor samples and cell lines using DNAzol^® ^genomic DNA isolation reagent (Molecular Research Center, Inc., Cincinnati, OH) following the manufacturer instructions. The DNA concentration was determined by measuring the 260/280 nm absorbance of each sample with a Pharmacia Biotech Ultrospec 3000 (Manufacturer name and address).

### Detection of MMTV-like gene sequences by PCR

PCR was performed using three set of primers to amplify different specific segments of the MMTV *env *gene. The 1 - 3 primers (5'-CCTCACTGCCAGATC-3', 5'-ATCTGTGGCATACCT-3') amplify a 665-bp segment and 2N-3N primers (5'-CCTACATCTGCCTGTGTTAC-3', 5'-ATCTGTGGCATACCTAAAGG-3') amplify a 254-bp segment. The 5L-3L primers (5'-CCAGATCGCCTTTAAGAAGG-3', 5'-TACAGGTAGCAGCACGTATG-3') were used to amplify a 595-bp fragment. To amplify a 1,338-bp segment of MMTV LTR*-gag*, we used the LTR5'-GAG 3 primers (5'-GGTGGCAACCAGGGACTAT-3', 5'-GACAGCTTGTCTACCTCTGT-3') and the LTR5'- LTR3' primers (5'-GGTGGCAACCAGGGACTTAT-3', CGAACAGACACAAACACACG-3') to amplify a 630-bp segment.

The env set primers were described by Wang et al. in 1995 [15]. The LTR5, LTR3, and GAG primers were described by Liu B et al. [18] PCR was performed in triplicate as previously described using standard PCR procedures to avoid contamination. DNA quality was assessed by amplifying a 452-bp fragment of the G3PDH gene using the following primers: forward 5`-ACCACAGTCCATGCCATCAC-3` and reverse 5`-TCCACCACCCTGTTGCTGTA`-3`. The amplified product**s **were analyzed by electrophoresis on a 1% agarose gel. Images were acquired, and analyses were performed using the gel imaging and analysis system (D&RI Ind. Ltd. Transilluminator and Gel-Pro Imager).

### DNA sequencing

The MMTV PCR products were ligated into pCR 4-TOPO (Invitrogen, Carlsbad, CA) and transfected into *E. Coli *(Top10 cells; Invitrogen). The cultures were grown overnight at 37°C in an LB agar plate. Positive colonies were selected and grown in LB broth. Isolation and purification of plasmids were performed with the Rapid Plasmid Purification Systems (Marligen Bioscience, Inc., Ijamsville, MD). To detect the cloned inserts, we performed an EcoRI digestion followed by 1.2% agarose electrophoresis. The positive sequences were analyzed and compared with previously reported MMTV-like sequences (AY161347) using BLAST [29]

## Competing interests

The authors declare that they have no competing interests

## Authors' contributions

Conceived and designed the experiments: TAL, ZBP. Performed experiments: MSK, GSJ, SAS. Analyzed the data: TAL, ZBP, SAS, CRP, TGR. Participated in the specimen and data collection and testing BRR, BAI. Wrote the paper: ZBP, ZAD, TAL. All authors read and approved the final manuscript.
